# The m^6^A Methylation-Regulated AFF4 Promotes Self-Renewal of Bladder Cancer Stem Cells

**DOI:** 10.1155/2020/8849218

**Published:** 2020-07-02

**Authors:** Qian Gao, Jin Zheng, Zegui Ni, Pengli Sun, Congcong Yang, Maosheng Cheng, Mingqing Wu, Xiuhong Zhang, Lin Yuan, Yingyin Zhang, Yang Li

**Affiliations:** ^1^Department of Genetics, School of Life Science, Anhui Medical University, Hefei, Anhui 230031, China; ^2^Department of Pathology, Shanghai General Hospital, Shanghai Jiaotong University School of Medicine, No. 100 Hai Ning Road, Hongkou District, Shanghai 200080, China

## Abstract

The dynamic N^6^-methyladenosine (m^6^A) modification of mRNA plays a role in regulating gene expression and determining cell fate. However, the functions of m^6^A mRNA modification in bladder cancer stem cells (BCSCs) have not been described. Here, we show that global RNA m^6^A abundance and the expression of m^6^A-forming enzyme METTL3 are higher in BCSCs than those in non-CSCs of bladder cancer (BCa) cells. The depletion of the *METTL3* inhibited the self-renewal of BCSCs, as evidenced by decreased ALDH activity and sphere-forming ability. Mechanistically, METTL3 regulates the m^6^A modification and thereby the expression of AF4/FMR2 family member 4 (AFF4), knockdown of which phenocopies the *METTL3* ablation and diminishes the tumor-initiating capability of BCSCs *in vivo*. AFF4 binds to the promoter regions and sustains the transcription of *SOX2* and *MYC* which have critical biological functions in BCSCs. Collectively, our results demonstrate the critical roles of m^6^A modification in self-renewal and tumorigenicity of BCSCs through a novel signaling axis of METTL3-AFF4-SOX2/MYC.

## 1. Introduction

Cancer stem cells (CSCs, also known as tumor-initiating cells), a relatively rare population of cancer cells, have characteristics of self-renewal capability, tumorigenic capacity, and pluripotency, which contribute to the driving force of tumorigenesis and metastasis. These stemness properties make CSCs resistant to conventional chemotherapies and cause subsequent recurrence, leading to clinical treatment failure [1]. Effective therapeutics and strategies targeting CSCs are desperately needed, whereas our knowledge of the CSCs is still incomplete so far.

Bladder carcinoma (BCa) is one of the most common malignancies and is characterized by rapid progression and high risk of recurrence [2, 3]. To better understand and eventually eliminate the bladder cancer stem cells (BCSCs), we and other groups have successfully identified several different BCSCs and determined their roles in BCa progression *in vivo* [4–7]. Moreover, we have found low-dose decitabine (a DNA methyltransferase inhibitor) could diminish the stemness of BCSCs without causing severe cytotoxicity [8], suggesting an important role of epigenetic regulation in BCSCs.

Besides the DNA methylation, recently we and others have found that aberrant N^6^-methyladenosine (m^6^A) methylation was also implicated in BCa progression [9–11]. RNA m^6^A is the most prevalent chemical mark observed in approximately 25% of eukaryotic mRNAs [12–14]. In mammalian cells, this dynamic modification is catalyzed by a methyltransferase complex consisting of several “writers,” which include methyltransferase-like 3 (METTL3), METTL14, Wilms tumor 1-associated protein (WTAP), VIRMA (KIAA1429), and RBM15 [15–19], and removed by two “erasers”: fat mass and obesity-associated protein (FTO) [20] and alkylation repair homolog protein 5 (ALKBH5) [21]. Aberrant m^6^A modification plays crucial roles in the progression of different types of cancer [22], especially as the modulator of CSCs of breast cancer [23], glioblastoma [24, 25], and leukemia [26, 27]. However, its function and mechanism in regulating CSCs seem to be context-dependent and have not been described in BCSCs so far.

In our previous study, we found that m^6^A abundance of both *MYC* and *AFF4* mRNAs was regulated by aberrantly expressed METTL3 in BCa cells [11]. As a core component of the super elongation complex (SEC), AFF4 is involved in the regulation of transcription elongation of many genes encoding the pluripotency factors [28, 29]. For instance, AFF4 could upregulate *SOX2* transcription to promote the tumor-initiation capacity of head and neck squamous cell carcinoma (HNSCC) [30], and *MYC* is another known target of AFF4 [11, 31]. Inspired by these results, we hypothesized that m^6^A plays a role in promoting the stemness of BCa cells by regulating AFF expression.

Here, we provide unequivocal evidences that the expression of METTL3 and RNA m^6^A level is significantly higher in the CSCs relative to the non-CSCs of BCa; METTL3 promote the self-renewal capability of BCSCs by regulating the mRNA m^6^A level and therefore the expression of AFF4, which in turn bind to the promoter regions of *SOX2* and *MYC* to activate their transcription. Our findings reveal the role and mechanism of RNA m^6^A in regulating the stemness of BCSCs and will inspire future studies regarding their applications in clinical treatment.

## 2. Materials and Methods

### 2.1. Cell Culture, Flow Cytometry, and Sphere Formation Assays

BCa cell lines 5637 (ATCC NO. HTB-9) and UM-UC-3 (ATCC NO. CRL-1749) were purchased from the Chinese Academy of Cell Resource Center (Shanghai, China) and maintained as previously described [32]. Cell lines were routinely tested for mycoplasma and not cultured for longer than 20 passages. Specific siRNAs were transfected into cells by Lipofectamine™ RNAiMAX Transfection Reagent (13778-075) according to the manufacturer's instructions.

For flow cytometry analysis, BCa cells were stained using the ALDEFLUOR assay kit (StemCell Technologies) according to the manufacturer's instructions. Acquisition and sorting were then performed using the BECKMAN Moflo XDP (Beckton Dickson, Mountain View, CA). Gates for fluorescence fractionations were established using unstained and isotype controls.

For sphere formation assays, FACS-sorted cells were cultured in 24-well ultralow attachment plates (Corning Inc., Corning, NY, USA) at a density of 1,000 cells per well. Cells were cultured in serum-free DMEM/F12 supplemented with growth factors EGF, *β*-FGF, and IGF-1 at a concentration of 20 ng/ml (PeproTech, Rocky Hill, NJ, USA). Spheres with a diameter of over 20 *μ*m were counted 7 days after plating.

### 2.2. Detect Gene Expression

For mRNA level examination, total RNA of BCa cells was extracted using Trizol reagent (Invitrogen). Complementary DNA (cDNA) synthesis was performed with the PrimeScript™ RT reagent Kit with gDNA Eraser (Takara, RR047A) using 1 *μ*g RNA per sample. qPCR reactions were performed using TB Green® Premix Ex Taq™ (Takara RR820A) to determine mRNA transcript levels. Primers for qRT-PCR are listed in Supplementary Table S1, siRNAs are used to knockdown METTL3, and AFF4 expression is listed in Supplementary Table S2.

For Western blotting, BCa cells were lysed with RIPA buffer as a standard protocol. The cell lysate was then mixed with loading buffer and incubated at 100°C for 5 min and subjected to conventional Western analysis. Antibodies are listed in Supplementary Table S3. The relative levels of proteins were quantified using densitometry with the Gel-Pro Analyzer (Media Cybernetics, Rockville, MD, USA). The target bands were densitometrically quantified and indicated under each band.

### 2.3. m^6^A Quantification

RNA m6A levels were evaluated by the m^6^A RNA quantification kit (Epigentek, P-9005) according to the manufacturer's protocol. Briefly, 200 ng total RNA of each sample was bound to the strip well of a 96-well plate, followed by m^6^A antibody capture and washing. After incubated with the substrate for 5 min before the reaction was stopped, the absorbance of each well was read on a microplate reader (Multiskan FC, Thermo Scientific) at 450 nm.

### 2.4. CHIP Assay

Chromatin immunoprecipitation (ChIP) assay was performed using a Simple ChIP Assay Kit (Cell Signaling Technology, Danvers, MA) according to the manufacturer's instruction. The precipitated DNA samples were purified and measured by qPCR. Results were shown as the percentage of input controls. Primers and antibodies used for CHIP assay are listed in the supplementary Table S1 and Table S2, respectively.

### 2.5. Limiting Dilution Transplantation Assay

Stable AFF4 knockdown 5637 cells and control cells were serially diluted (1 × 10^5^-2.7 × 10^6^), resuspended in 50 *μ*l of Matrigel (Corning, 354230), and injected subcutaneously into BALB/cASlac-nu nude mice (Shanghai Laboratory Animals Center, SLAC). Subsequent tumors were monitored weekly until mice presented signs of distress, and the mice were sacrificed. All animal procedures were performed under a protocol approved by the Laboratory Animal Center of Anhui Medical University.

Paraffin sections of samples from xenografts were antigen retrieved, blocked, and processed as described before [33]. The intensity of immunostaining was measured by Image-Pro Plus 6.0 image analysis software (Media Cybernetics). The intensity of each image was calculated by normalizing the average integrated optical density (IOD) with the total selected area of interest (AOI).

### 2.6. Statistics

All experiments were performed at least three times, unless otherwise noted. Data are presented as the means ± standard deviation (S.D.) or standard error (S.E.). All of the statistical analyses were performed using Excel (Microsoft, Redmond, WA) or Prism (GraphPad Software Inc., La Jolla, CA). The two-tailed Student's *t*-test was used, and a *p* value of <0.05 was considered significant. For limiting dilution assay, a statistical test was performed as described previously [34].

## 3. Results

### 3.1. RNA m^6^A Levels Are Elevated in BCSCs

**Figure 1:**
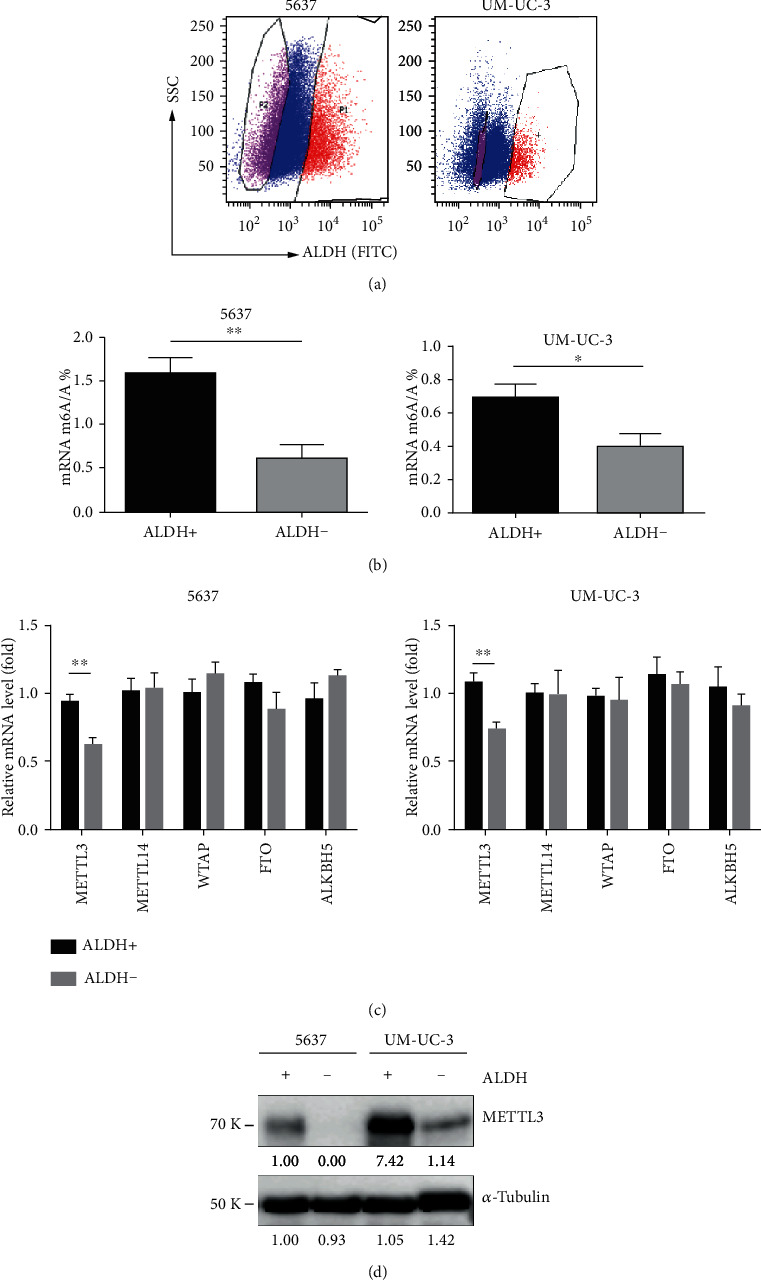
Differential m^6^A levels between CSCs and non-CSCs of BCa. (a) Representative gating scheme for FACS sorting of ALDH1-stained 5637 and UM-UC-3 cells. (b) Quantification of m^6^A levels in ALDH1-positive and ALDH1-negative BCa cells (^∗^*p* < 0.05, ^∗∗^*p* < 0.01, Student *t*-test). (c) mRNA levels of RNA m^6^A writers and erasers in ALDH1-positive and ALDH1-negative BCa cells (^∗∗^*p* < 0.01, Student *t*-test). (d) Protein levels of RNA m^6^A methyltransferase METTL3 in ALDH1-positive and ALDH1-negative BCa cells.

To estimate the potential role of RNA m^6^A modification in regulating the stemness of BCa, we examined the global RNA m^6^A levels of CSCs and non-CSCs of BCa. Aldehyde dehydrogenase 1 family, member A1 (ALDH1A1) was used as a marker to isolate BCSCs [35] from two established cancer cell lines, 5637 and UM-UC-3, by flow cytometry (Figure 1(a)), and RNA m^6^A methylation abundance was evaluated by the m^6^A RNA quantification kit. The results showed that ratios of m^6^A RNA/total RNA in ALDH1-positive (ALDH1^+^) cells isolated from both 5637 and UM-UC-3 were significantly higher than those in ALDH-negative (ALDH1^−^) proportion (Figure 1(b)). We then checked the expression patterns of known m^6^A writers (i.e., *METTL3*, *METTL14*, and *WTAP*) and erasers (i.e., *FTO* and *ALKBH5*) by quantitative RT-PCR to determine which subunit may account for m^6^A dysregulation of CSCs and found that the expression of *METTL3* rather than other regulators was significantly elevated in ALDH1^+^ BCa cells (Figure 1(c)). The protein level of METTL3 was further validated to be higher in ALDH1^+^ BCa cells by Western blot (WB) (Figure 1(d)). All these data indicate that METTL3 is upregulated in BCSCs and may be implicated in self-renewal.

### 3.2. Targeting METTL3 Expression Impairs BCSC Self-Renewal

**Figure 2:**
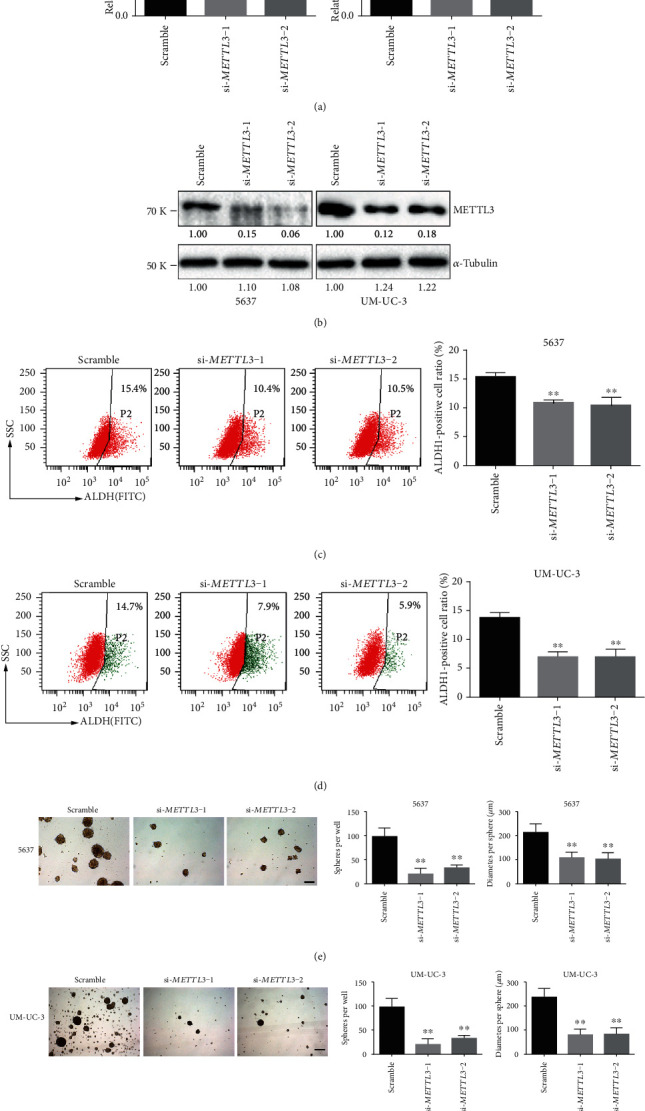
METTL3 is required to sustain self-renewal of BCa cells. The knockdown effect of specific siRNAs (si-METTL3-1 and si-METTL3-2) in 5637 and UM-UC-3 cells was verified at both the mRNA ((a) by qRT-PCR) and protein levels ((b) by Western blot). Ratio of cells with high ALDH activity (c, d); number and size of spheres formed in stem cell medium (e, f) of the BCa cells transfected with indicated siRNAs are plotted, and representative images are presented. ^∗∗^*p* < 0.01 compared to the scramble group, by Student *t*-test. Scale bar, 250 *μ*m.

To determine whether METTL3 is important to BCSC self-renewal, we used two distinct siRNAs (si-METTL3-1 and si-METTL3-2) to ablate METTL3 expression in 5637 and UM-UC-3 cells. Compared with a nontargeting control siRNA (si-GFP), both specific siRNAs significantly reduced METTL3 mRNA and protein levels (Figures 2(a) and 2(b)). Two characteristics to identify populations of BCSCs are the ability to generate clusters of daughter cells when they are cultured on ultralow adherence plates (sphere assay) and high ALDH activity which can be quantified by flow cytometry using a fluorogenic substrate [7].

36 hours after siRNA transfection, cells with high ALDH activity were examined and sorted by flow cytometry, and the same amount of cells with high ALDH activity from different transfection groups was further transferred to ultralow adherence plates with stem cell medium. One week later, the number of formed spheres was counted. Both the percentages of cells with high ALDH activity (Figures 2(c) and 2(d)) and sphere formation frequency (Figures 2(e) and 2(f)) were significantly decreased upon *METTL3* knockdown. With the above evidences, we concluded that METTL3 is required for the BCSC self-renewal *in vitro*.

### 3.3. AFF4 Is Regulated by METTL3 in BCSCs

**Figure 3:**
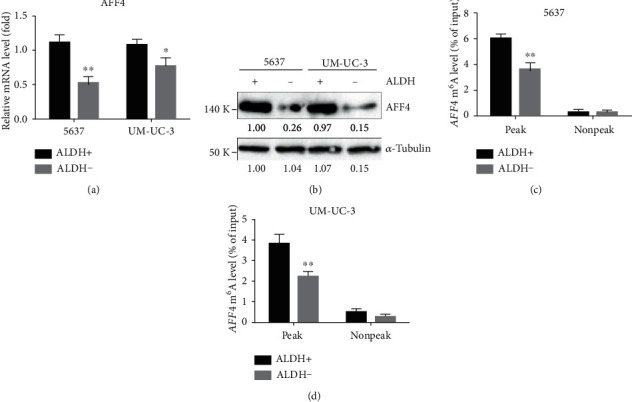
Differential expression level and m^6^A levels of *AFF4* between CSCs and non-CSCs of BCa. mRNA levels (a) and protein levels (b) of AFF4 in ALDH1-positive and ALDH1-negative BCa cells. m^6^A modification in specific regions of *AFF4* transcripts in ALDH1-positive and ALDH1-negative 5637 (c) and UM-UC-3 (d) cells was tested by gene-specific m^6^A-qPCR assay. ^∗^*p* < 0.05, ^∗∗^*p* < 0.01 compared to the scramble group, by Student *t*-test.

**Figure 4:**
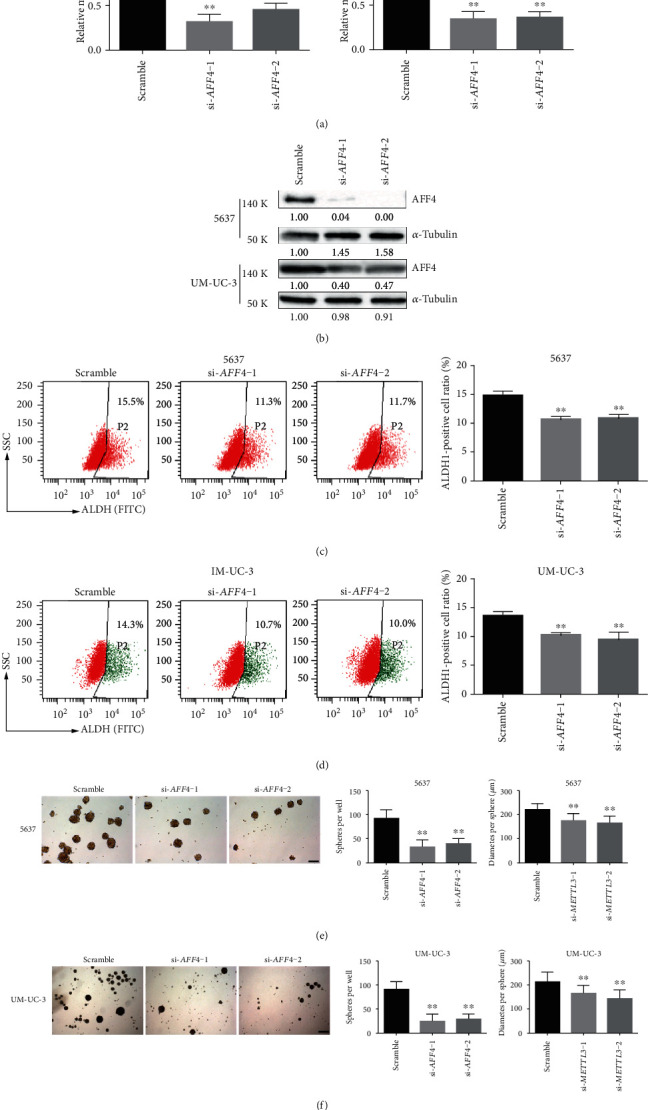
*AFF4* mimic the phenotype of *METTL3* in regulating the stemness of BCSCs. The knockdown effect of specific siRNAs (si-*AFF4*-1 and si-*AFF4*-2) in 5637 and UM-UC-3 cells was verified at both the mRNA ((a) by qRT-PCR) and protein levels ((b) by Western blot). Ratio of cells with high ALDH activity (c, d); number and size of spheres formed in stem cell medium (e, f) of the BCa cells transfected with indicated siRNAs are plotted, and representative images are presented. ^∗^*p* < 0.05, ^∗∗^*p* < 0.01 compared to the scramble group, by Student *t*-test. Scale bar, 250 *μ*m.

In the previous study, we performed transcriptome sequencing and m^6^A sequencing followed by a series validation in 5637 cells, which proved the m^6^A modification and expression of *AFF4* mRNA were directly regulated by METTL3 [11]. To identify if AFF4 was also the target of METLL3 in BCSCs, we then checked both mRNA and protein levels of AFF4 in ALDH1^+^ and ALDH1^−^ cells from 5637 and UM-UC-3, respectively. Not surprisingly, a significantly higher level of AFF4 expression was observed in the ALDH1^+^ proportion compared to the corresponding ALDH1^−^ counterpart in BCa cells (Figures 3(a) and 3(b)). Moreover, gene-specific m^6^A-qPCR using primers to amplify either the m^6^A peak region (indicated by our m^6^A-sequencing results) or a control (non-peak) region showed a markedly increased m^6^A abundance of *AFF4* mRNA in ALDH1^+^ BCa cells (Figures 3(c) and 3(d)), which suggest the difference of AFF4 expression between CSCs and non-CSCs is regulated by METTL3-mediated m^6^A modification primarily. To validate if *AFF4* acted downstream of METTL3 in BCSCs, we further analyzed the effect of AFF4 deficiency on the BCa self-renewal using a similar strategy to METTL3 knockdown. With effective ablation of AFF4 expression by siRNAs in both 5637 and UM-UC-3 cells (Figures 4(a) and 4(b)), both ALDH activity (Figures 4(c) and 4(d)) and sphere formation frequency (Figures 4(e) and 4(f)) showed a significant decrease upon *AFF4* knockdown, which mimic the phenotype resulting from METTL3 knockdown and indicate the regulatory relationship between AFF4 and METTL3 in BCSC self-renewal.

### 3.4. AFF4 Directly Regulates *MYC* and *SOX2* Gene Expression in BCa Cells

**Figure 5:**
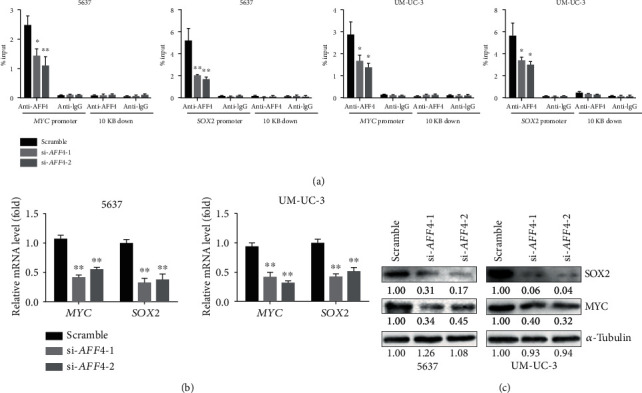
AFF4 regulates SOX2 and MYC expression in BCa cells. (a) ChIP assay showed the recruitment of AFF4 at *MYC* and *SOX2* promoter regions in 5637 and UM-UC-3 cells transfected with indicated siRNAs at 48 h posttransfection. Expression of MYC and SOX2 in 5637 and UM-UC-3 cells transfected with indicated siRNAs (si-*AFF4*-1 and si-*AFF4*-2) was verified at both the mRNA ((b) by qRT-PCR) and protein levels ((c) by Western blot). ^∗^*p* < 0.05, ^∗∗^*p* < 0.01 relative to the scramble group by Student *t*-test.

As an essential component of SEC, AFF4 can bind to DNA directly and regulate the transcription elongation of many genes. MYC and SOX2, well-known pluripotency factors of CSCs, have been reported to be regulated by AFF4 in BCa [11] and HNSCC [30], respectively. To investigate whether MYC and SOX2 are effectors of AFF4 in regulating the self-renewal capability of BCSCs, we performed CHIP assay in 5637 and UM-UC-3 cells and found AFF4 directly bound to *MYC* and *SOX2* promoter regions, which were barely detectable after *AFF4* knockdown (Figure 5(a)). Besides, we also confirmed the expression of MYC and SOX2 in response to *AFF4* knockdown by qRT-PCR and Western blot. The results showed knockdown of *AFF4* drastically reduced the expression of these two genes at both mRNA level and protein level (Figures 5(b) and 5(c)).

### 3.5. AFF4 Promotes BCSC Self-Renewal *In Vivo* and Is a Negative Prognostic Factor for BCa Patients

**Figure 6:**
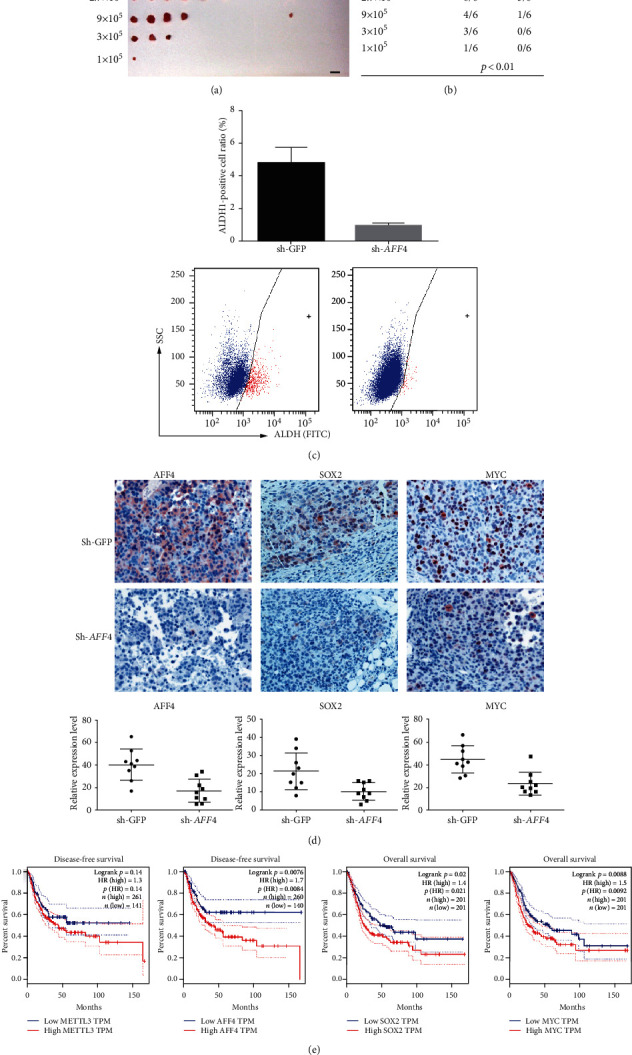
AFF4 is essential for BCa tumor propagating *in vivo*. Graph (a) and quantification (b) of the percentage of tumor-free mice 30 days after subcutaneous injection of different dilutions of AFF4 knockdown 5637 cells or control cells into immunodeficient mice (*n* = 6 for each dilution). (c) Ratio of ALDH-positive cells from the xenografts. ^∗∗^*p* < 0.01 by Student *t*-test. (d) Quantitative measurement and representative images of AFF4, SOX2, and MYC expression in xenografts generated by AFF4 stable knockdown BCa cells and control cells. (^∗∗^*p* < 0.01 by Student *t*-test). Scale bar, 50 *μ*m. (e) Correlation between METTL3, AFF4, SOX2, and MYC mRNA expression and survival of BCa patients in TCGA dataset. Disease-free or overall patient survival in groups of high and low expression was analyzed by the Kaplan-Meier survival curve and compared by the log-rank test.

To further evaluate the effect of *AFF4* depletion on the self-renewal capacity of BCSCs *in vivo*, we conducted limiting dilution transplantation assay, a method widely used to assess cancer stem cell content. *AFF4* expression was stably ablated by short hairpin RNA (sh-AFF4) in 5637 cells, which were then injected subcutaneously into immune-deficient mice, and tumor growth was measured over time. Consistent with the *in vitro* results, AFF4-deficient cells exhibited a significantly lower tumor-propagating potential than the control cells (sh-GFP) comprising the tumor bulk (Figures 6(a) and 6(b)). Following ALDH1 staining and FACS analysis showed a clear reduction of ALDH-positive ratio (Figure 6(c)), along with AFF4, SOX2, and MYC expression in xenografts generated from sh-AFF4 5637 cells relative to the control tumors (Figure 6(d)).

The cancer stemness properties of BCSCs contribute to the chemoresistance, metastasis, and recurrence, which are often related to poor clinical outcome. We queried The Cancer Genome Atlas (TCGA) database and analyzed the survival curve of BCa with the help of the GEPIA online tool [36]. A worse disease-free survival was found in the METTL3 high expression group than that in the METTL3 low expression group, and the *p* value shows a certain trend toward significance (Figure 6(e), *p* = 0.11), while higher expression of *AFF4* was clearly a significant indicator of poor prognosis of BCa (Figure 6(e), *p* = 0.008). Besides, the higher expression of SOX2 (Figure 6(e), *p* = 0.02) and MYC (Figure 6(e), *p* = 0.009) was also significantly associated with worse overall survival. Taken together, aberrant expression of AFF4 is associated with BCSCs within the tumor bulk which may lead to poor prognosis.

## 4. Discussion

We have shown in our previous study that METTL3 plays a critical role in the pathogenesis of BCa, by positively regulating the expression of IKBKB, RELA, AFF4, and MYC through m^6^A-based posttranscriptional regulation [11]. Here, we demonstrate that mRNA m^6^A modification is critical for maintaining BCSC self-renewal and tumor development. The knockdown of *METTL3* expression reduced the self-renewal of BCSCs. Emerging data have suggested that the global abundance of m^6^A and expression levels of its regulators, including writers, erasers, and readers, are often dysregulated in various types of cancers and are critical for cancer initiation, progression, metastasis, and drug resistance and cancer relapse [22]. Intriguingly, reasons of m^6^A dysregulation in CSCs are different among various types of cancer, considering the roles of FTO, ALKBH5, and METTL3 in glioblastoma stem cells [24, 25] and of METTL14 and FTO in leukemia stem cells [26, 27]. In BCa, our data shows METTL3 is the only regulator that is aberrantly expressed and critical for BCa pathogenesis and BCSC maintenance. This study uncovered a critical role of mRNA m^6^A modification in regulating BCSCs self-renewal and tumorigenesis. Nevertheless, the reason for aberrant *METTL3* expression in BCa is still unknown and awaits further investigation.

AFF4 is a core component and required for SEC stability and activity, by acting as a scaffold to assemble the SEC [37, 38]. Evidences showing that AFF4 might play a role in regulating pluripotency include its involvement in the osteogenic differentiation of human mesenchymal stem cells [28] and odontogenic differentiation of human dental pulp cells [29]. AFF4 is also required for the tumor-initiating capacity of stem-like cells in HNSCC [30]. In our previous study, AFF4 was indicated by our transcriptome and m^6^A sequencing data to be a direct target of METTL3 in BCa cells; we then demonstrated that *AFF4* mRNA is regulated by METTL3 in a m^6^A-dependent manner [11]. In the current study, we reveal that both the m^6^A abundance and the expression level of *AFF4* mRNA are elevated in BCSCs, which is consistent with the expression pattern of METTL3. Moreover, ALDH activity and sphere-forming ability *in vitro* as well as tumor-initiating capacity *in vivo* were all abrogated upon *AFF4* knockdown. Besides, there was a clear correlation between AFF4 expression and BCa invasion potential [11], which is another commonly used indicator of tumorigenicity. Taken together, our data suggest AFF4 is a *bona fide* target of METTL3 in regulating the self-renewal capacity of BCSCs.

Our previous work proved Sox2 as a marker for stem-like tumor cells of BCa *in vivo* [7]. Besides, there are evidences indicated that downregulation of c-Myc suppressed CSC differentiation in BCa, and overexpression of c-Myc increased the levels of stem cell markers including SOX2 [39]. Therefore, SOX2 and MYC both are master regulators of self-renewal and differentiation of CSCs and are essential for BCa initiation and progression. *SOX2* mRNA was reported to contain m^6^A modification in embryonic stem cells [40] and glioblastoma stem cells [24], and m^6^A modification of *MYC* mRNA was found in the CSCs of acute myeloid leukemia. Indeed, we have also confirmed MELLT3 could regulate MYC expression by promoting the m^6^A modification of its mRNA in BCa cells [11]. It is likely that METTL3 promote the expression of SOX2 and MYC through m^6^A-based posttranscriptional regulation as well as AFF4-mediated regulation at the transcriptional level, which reinforces the signal activating the tumor-initiating and self-renewal capabilities of BCa cells. Meanwhile, methyltransferase METTL3 has a global effect on many RNAs; just like AFF4/SEC, MYC and SOX2 exert a broad effect on the expression of various pluripotency-related genes by binding to multiple sites of DNA. Therefore, the role of METTL3 regulating BCSCs might not merely rely on AFF4. Other potential target genes involved in BCa initiation and self-renewal need to be investigated.

In summary, we found m^6^A modification of *AFF4* RNA was upregulated by METTL3 and their expression was elevated in BCSCs, which in turn promotes the expression of SOX2 and MYC to enhance tumorigenesis and tumor-initiating capacity of BCa. Our findings indicate AFF4 may serve as a biomarker and a potential target of therapies for patients with BCa.

## Data Availability

All data is available upon request by contacting the corresponding authors: Yang Li, Ph.D. Department of Genetics, School of Life Science, Anhui Medical University, Hefei, Anhui 230031, China; Tel.: 86 551-65160327; E-mail: liyang@ahmu.edu.cn; and Yingyin Zhang, Department of Genetics, School of Life Science, Anhui Medical University, Hefei, Anhui 230031, China; Tel.: 86 551-65169646; E-mail: liyang@ahmu.edu.cn.

## Supplementary Materials

Supplementary Materials Supplementary Table 1: information of primers. Supplementary Table 2: information of siRNAs. Supplementary Table 3: information of antibodies. Supplementary Figure 1: backbone of the plasmids used for shRNA construct.
