# Supplementary material for: The m6A Methylation-Regulated AFF4 Promotes Self-Renewal of Bladder Cancer Stem Cells
Source: Stem Cells Int. 2020 Jul 2;2020:8849218. doi: 10.1155/2020/8849218 (PMC7352121; doi:10.1155/2020/8849218)
Supplement: Supplementary Materials — Supplementary Table 1: information of primers. Supplementary Table 2: information of siRNAs. Supplementary Table 3: information of antibodies. Supplementary Figure 1: backbone of the plasmids used for shRNA construct. [file 8849218.f1.docx]

**Supplementary Table1 Information of Primers**

|  | Primer Names | Sequences |
| --- | --- | --- |
| ForqRT PCR | hMETTL3 F | 5′-CAAGCTGCACTTCAGACGAA-3′ |
|  | hMETTL3 R | 5′-GCTTGGCGTGTGGTCTTT-3′ |
|  | hMETTL14 F | 5′-GAGTGTGTTTACGAAAATGGGGT-3′ |
|  | hMETTL14 R | 5′-CCGTCTGTGCTACGCTTCA-3′ |
|  | hWTAP F | 5′-ACTGGCCTAAGAGAGTCTGAAG-3′ |
|  | hWTAP R | 5′-GTTGCTAGTCGCATTACAAGGA-3′ |
|  | hALKBH5 F | 5′-CCTGAGCAGCTCCGTGTTAC-3′ |
|  | hALKBH5 R | 5′-CCAGGATCCGTGGCCTGTG-3′ |
|  | hFTO F | 5′-CTGTGAAGGCCCTGAAGAGG-3′ |
|  | hFTO R | 5′-AAGGGGTATCGCCAAACCAG-3′ |
|  | hMYC F | 5′-TTCGGGTAGTGGAAAACCAG-3′ |
|  | hMYC R | 5′-AGTAGAAATACGGCTGCACC-3′ |
|  | hAFF4 F | 5′-AAAGGCCAGCATGGATCAGAA-3′ |
|  | hAFF4 R | 5′-GTGATTTGGAGCGTTGATGTTC-3′ |
|  | hSOX2 F | 5’-GCGAACCATCTCTGTGGTCT-3’ |
|  | hSOX2 R | 5’-GGAAAGTTGGGATCGAACAA-3’ |
|  | hGAPDH F | 5'-AGGTCGGTGTGAACGGATTTG -3' |
|  | hGAPDH R | 5'-GGGGTCGTTGATGGCAACA -3' |
| m6A-qPCR | MYC-m6A-F | 5′-GCATACATCCTGTCCGTCCA-3′ |
|  | MYC-m6A-R | 5′-GTCGTTTCCGCAACAAGTCC-3′ |
|  | AFF4-m6A-F | 5′-GGAATAGTTTTCTCCTCGTTGGTTC-3′ |
|  | AFF4-m6A-R | 5′-ATCAATGTGCTGCAGATTTAAAAGC-3′ |
| CHIP-qPCR | MYC promoter F | 5′-GCAATGCGTTGCTGGGTTATT-3′ |
|  | MYC promoter R | 5′-GCGTTCAGAGCGTGGGATGTT-3′ |
|  | MYC 10KB F | 5′-AATTGCCTGCATTCACAGAGC-3′ |
|  | MYC 10KB R | 5′-TCCATCCCATTTATCTTGGTATCT-3′ |
|  | SOX2 promoter F | 5′-CGTCACATGGATGGTTGTCTAT-3′ |
|  | SOX2 promoter R | 5′-GGCTCAAACTTCTCTCCCTTTC-3′ |
|  | SOX2 10KB F | 5′-TGGTGCAAAAACATCTTGGA-3′ |
|  | SOX2 10KB R | 5′- TACCCAAGAACCAGGAGTGG-3′ |

**Supplementary Table2 Information of siRNAs**

| siRNAs | Sequence |
| --- | --- |
| si-METTL3-1 | F:5‘-GCUGCACUUCAGACGAAUUTT-3’  R:5‘-AAUUCGUCUGAAGUGCAGCTT-3’ |
| si-METTL3-2 | F:5’-CUGCAAGUAUGUUCACUAUGA-3’  F:5’-UCAUAGUGAACAUACUUGCAG-3’ |
| si-AFF4-1 | F:5‘-GUUACUAGCAAAGAAGAUATT-3’  R:5‘-UAUCUUCUUUGCUAGUAACTT-3’ |
| si-AFF4-2 | F:5‘-GUAUGCUUGGAAACUACGATT-3’  R:5‘-UCGUAGUUUCCAAGCAUACTT-3’ |

**Supplementary Table3 Information of Antibodies**

| Protein target | Company | Catalog Number | Host species | Clonality |
| --- | --- | --- | --- | --- |
| METTL3 | Protein Tech | 15073-1-AP | Rabbit | polyclonal |
| MYC | Protein Tech | 10828-1-AP | Rabbit | polyclonal |
| SOX2 | Protein Tech | 11064-1-AP | Rabbit | polyclonal |
| AFF4 | Protein Tech | 14662-1-AP | Rabbit | polyclonal |
| α-Tubulin | Protein Tech | 11224-1-AP | Rabbit | polyclonal |


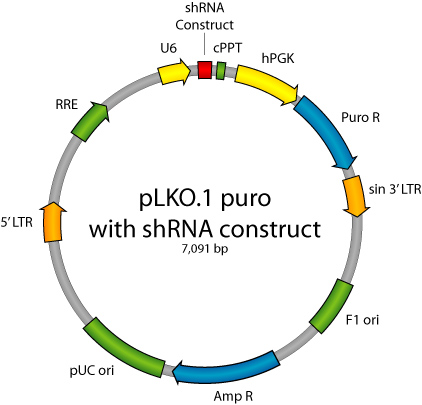


**Supplementary Figure 1, Backbone of the plasmids used for shRNA construct**

(From: [www.addgene.org](http://www.addgene.org))
